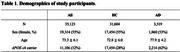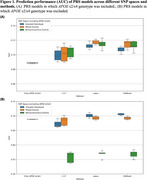# Impact of SNP Space on Polygenic Risk Modeling for Alzheimer’s Disease

**DOI:** 10.1002/alz70861_108637

**Published:** 2025-12-23

**Authors:** Junyoung Park, Zihuai He, Michael D. Greicius

**Affiliations:** ^1^ Department of Neurology and Neurological Sciences, Stanford University School of Medicine, Stanford, CA USA

## Abstract

**Background:**

Polygenic risk scores (PRS) are widely used to predict Alzheimer’s disease (AD) risk. Most models use millions of genome‐wide SNPs, including many in non‐coding regions. However, this can increase computational cost and reduce interpretability, with limited gains in predictive performance. In this study, we assessed whether narrowing down the SNP space to more biologically interpretable SNP spaces (whole exome or non‐synonymous exonic variants) can still give us reasonable predictive power.

**Method:**

We analyzed 35,123 European ancestry participants from the UK Biobank, consisting of 31,604 age‐matched healthy controls and 3,519 AD patients (Table 1). PRS performance was compared across three SNP spaces after QC (HWE *p* < 10^‐6^, MAF > 0.01): (1) imputed (∼5.4M SNPs), (2) whole exome (∼90K), and (3) non‐synonymous exonic variants (∼28K). GWAS was conducted for each space, and three modelling methods— Clumping and Thresholding (C+T), Lasso regression, and Extreme Gradient Boosting (XGBoost)—were applied using resulting summary statistics. A 5‐fold cross‐validation (CV) framework was used with training/validation/test splits. SNPs were selected using *p* ‐value thresholds from 10^‐8^ to 0.05, and the best model was chosen based on validation AUC and subsequently evaluated on the test set.

**Result:**

A model using only *APOE* ε2/ε4 genotype served as a baseline (AUC of 0.68–0.69.). Across all methods, PRS models based on whole exome or non‐synonymous SNPs showed comparable accuracy to those using imputed SNPs, despite far fewer variants (Figure 1A). Notably, when *APOE* ε2/ε4 genotype was excluded from PRS modelling, performance declined most sharply in models based on non‐synonymous exome variants, while whole exome–based models retained accuracy comparable to genome‐wide models (Figure 1B).

**Conclusion:**

Our findings underscore the potential of using biologically relevant SNP subsets to simplify models while maintaining performance. They also indicate that future PRS models for AD can be effectively developed using whole exome SNPs, providing a practical balance between predictive power and model simplicity. Taken together, these results support the use of more focused variant sets in cases where genome‐wide approaches may be computationally intensive or unnecessarily complex.